# By-Products from Food Industry as a Promising Alternative for the Conventional Fillers for Wood–Polymer Composites

**DOI:** 10.3390/polym13060893

**Published:** 2021-03-14

**Authors:** Aleksander Hejna, Jerzy Korol, Paulina Kosmela, Anton Kuzmin, Adam Piasecki, Arkadiusz Kulawik, Błażej Chmielnicki

**Affiliations:** 1 Department of Polymer Technology, Gdańsk University of Technology, Narutowicza 11/12, 80-233 Gdańsk, Poland; paulina.kosmela@pg.edu.pl; 2 Department of Material Engineering, Central Mining Institute, Pl. Gwarków 1, 40-166 Katowice, Poland; jkorol@gig.eu (J.K.); akulawik@gig.eu (A.K.); 3 Mechanics and Power Engineering Institute, Ogarev Mordovia State University, 68 Bolshevistskaya Street, Saransk 430005, Russia; kuzmin.a.m@yandex.ru; 4 Institute of Materials Engineering, Poznan University of Technology, Piotrowo 3, 61-138 Poznan, Poland; adam.piasecki@put.poznan.pl; 5 Paint & Plastics Department in Gliwice, Institute for Engineering of Polymer Materials and Dyes, 50 A Chorzowska Street, 44-100 Gliwice, Poland; b.chmielnicki@impib.pl

**Keywords:** wood–polymer composite, coffee silverskin, wood flour, brewers’ spent grain, mechanical properties, thermal properties

## Abstract

The present paper describes the application of two types of food-industry by-products, brewers’ spent grain (BSG), and coffee silverskin (ŁK) as promising alternatives for the conventional beech wood flour (WF) for wood–polymer composites. The main goal was to investigate the impact of partial and complete WF substitution by BSG and ŁK on the processing, structure, physicochemical, mechanical, and thermal properties of resulting composites. Such modifications enabled significant enhancement of the melt flowability, which could noticeably increase the processing throughput. Replacement of WF with BSG and ŁK improved the ductility of composites, which affected their strength however. Such an effect was attributed to the differences in chemical composition of fillers, particularly the presence of proteins and lipids, which acted as plasticizers. Composites containing food-industry by-products were also characterized by the lower thermal stability compared to conventional WF. Nevertheless, the onset of decomposition exceeding 215 °C guarantees a safe processing window for polyethylene-based materials.

## 1. Introduction

One of the environmentally friendly trends in polymer technology is incorporating by-products from various branches of industry as raw materials or intermediates in different processes. The food industry should be considered a sector which can significantly contribute due to the enormous amounts of generated waste [1]. Currently, around 1.6 billion tons of “primary product equivalents” is wasted each year globally [2]. Currently, food waste is mainly subjected to fermentation and used to produce biogas. Such a solution enables the partial energy recovery but does not utilize the full potential of compounds present in these materials. Therefore, it is vital to look for the possibilities for, mostly, reduction of the number of generated by-products as well as their utilization.

Multiple by-products generated by the food industry could be efficiently applied in polymer technology. These applications may be divided into two main groups: indirect and direct. The first group is mostly related to the extraction of chemical compounds from food by-products or their transformation into intermediates further used in polymer technology [3]. Extraction can be associated with particular components of materials such as cellulose, lignin, or oils but also specified compounds showing, among others, antioxidant, antimicrobial, or antifungal activity [4,5,6]. The second indirect approach includes pyrolysis, microbial treatment, or liquefaction of different types of widely understood biomass, aimed at polyols, for example, for the production of polyurethanes and epoxy resins or monomers for polymer production [7,8,9].

Direct applications of food-industry by-products in polymer technology are mostly related to their use as fillers for polymer composites, eventually after their surface treatment and large size reduction [10,11]. Such an approach is associated with their relatively similar chemical composition compared to other types of lignocellulose materials introduced into polymer composites, mostly wood flour [12]. Moreover, except for cellulose, hemicellulose, and lignin, which are by far the most significant wood-flour components, by-products from the food industry may also contain noticeable amounts of lipids and proteins [13,14]. These compounds may provide additional features to the fillers and composite materials. They may act as plasticizers, but also due to the presence of multiple functional groups may enhance the compatibility of fillers with polar polymer matrices [15].

The introduction of food-industry by-products into polymer technology may also reduce the use of synthetic compounds, which is in line with the current environmentally-friendly trends present in the industry [16]. Moreover, incorporating raw materials from natural sources may enhance the biodegradability of polymer materials [17].

Considering the issues mentioned in the above-presented research work aimed to investigate the possibility of partial substitution (from 0 to 100%) of conventional wood flour applied as a filler for wood–polymer composites with two types of by-products generated by the food industry: coffee silverskin and brewers’ spent grain. The impact of applied modifications on the processing (melt flow index), physicomechanical (density, static, and dynamic mechanical properties), and thermal (melting and crystallization behavior, and oxidation induction time) performance of composites based on waste low-density polyethylene (LDPE) was analyzed.

## 2. Materials and Methods

### 2.1. Materials

Recycled low-density polyethylene (LDPE), obtained from the local recycling company (Katowice, Poland), was applied as a matrix to prepare investigated composites. It was characterized by the density of 0.9142 g/cm^3^ and melt flow rate of 1.35 g/10 min (190 °C, 2.16 kg). Beech wood flour (WF) was obtained as a by-product from local furniture company (Kuków, Poland). Coffee silverskin (ŁK) was obtained from the industrial roastery (Marki, Poland) in the form of a pellet. Before introducing it into polyethylene, it was dried at 70 °C until a constant weight and grounded using Mockmill 200 stone grain mill (Wolfgang Mock Gmbh, Germany). Brewers’ spent grain (BSG) was obtained from Energetyka Złoczew Sp. z o.o. (Lututów, Poland). It was the waste from the production of light lager and consisted solely of barley malts. The supplier had already dried the obtained BSG. Before use, BSG applied in the presented study was ground in EHP 2 × 20 Sline co-rotating twin-screw extruder from Zamak Mercator (Skawina, Poland) as described in our patent application [18] and previous work [19].

**Figure 1:**
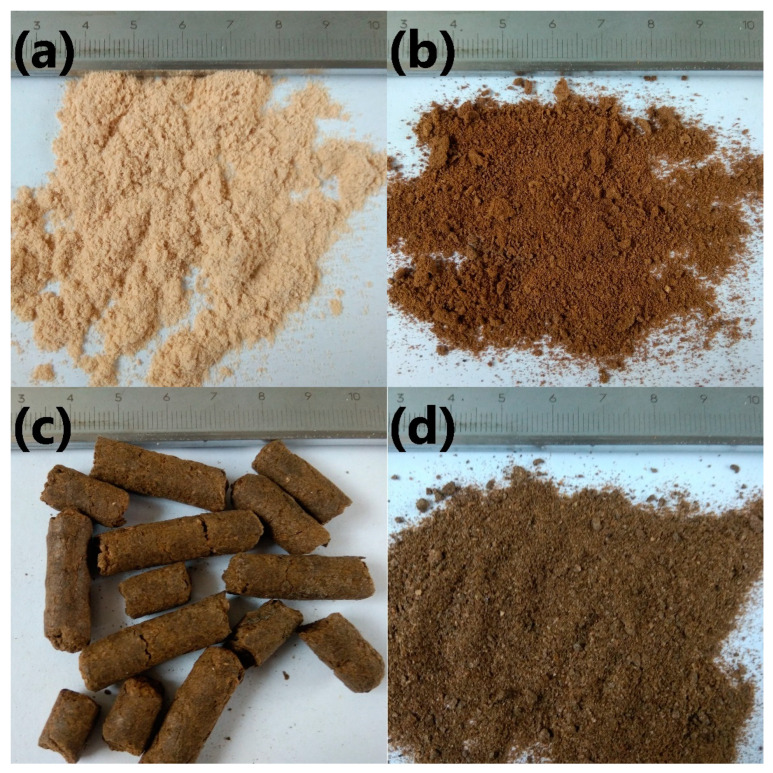
The appearance of the (**a**) beech wood flour, (**b**) modified brewers’ spent grain, as well as coffee silverskin (**c**) before and (**d**) after grinding.

**Figure 2:**
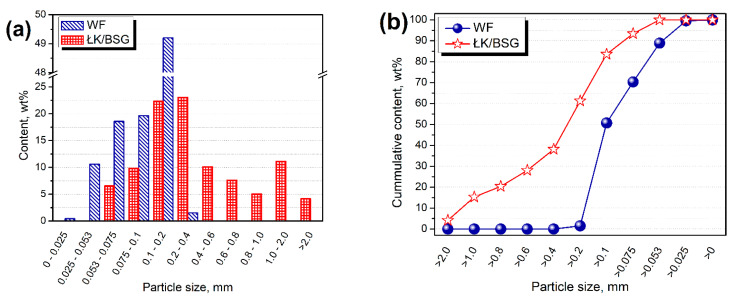
Plots of (**a**) particle size distribution and (**b**) cumulative particle size distribution of applied fillers.

In Figure 1, there are shown photographs of applied fillers. Figure 2 presents the particle size distribution of applied fillers. For a more effective comparison of two food-industry by-products, the particle size distribution of coffee silverskin was engineered to match brewers’ spent grain.

Moreover, for a more detailed analysis of the impact of food-industry by-products on prepared composites’ properties, chemical compositions of applied fillers based on the literature reports [20,21,22,23] are presented in Table 1.

### 2.2. Preparation of Polymer Composites

The composites were prepared by mixing in a molten state using a two-roll mill from Shaw Robinson (London, UK) at a temperature of 95 °C. Time of processing equaled 15 min, including the 3 min phase of polyethylene plasticization and 12 min of melt blending of polymer matrix with selected filler. The resulting composites were then compression molded at 150 °C and 4.9 MPa for 2 min and then kept under pressure at room temperature for another 5 min to enable solidifying the material. The detailed composition of samples is presented in Table 2.

### 2.3. Measurements

Melt flow index (MFI) of the composites was investigated using Zwick mFlow plastometer from Zwick (Ulm, Germany) according to ASTM D1238 standard (190 °C, 10 kg). A load of 10 kg was applied due to the low flowability of composites.

The specific weight of applied fillers and resulting composites was determined using Ultrapyc 5000 Foam gas pycnometer from Anton Paar (Graz, Austria). The following measurement settings were applied: gas—helium; target pressure—10.0 psi (for filler) and 18.0 psi (for composites); flow direction—sample first; temperature control—on; target temperature—20.0 °C; flow mode—fine powder (for filler) and monolith (for composites); cell size—small, 10 cm^3^; preparation mode—pulse, 5 pulses (for filler) and flow, 0.5 min (for composites); the number of runs—5.

The results obtained from pycnometry measurements were used to determine the porosity of composites as the difference between theoretical and experimental values of density. Theoretical values were calculated according to Formula (1):

ρ_theo_ = ρ_m_ · (1 − φ) + ρ_f_ · φ (1)

where ρ_theo_—theoretical density of the composite, g/cm^3^; ρ_m_—density of the matrix—0.9142 g/cm^3^; ρ_f_—density of the filler—1.4603, 1.4209, and 1.4041 g/cm^3^, respectively, for WF, ŁK, and BSG; and φ—a volume fraction of the filler.

To quantitatively determine the composite’s porosity, Equation (2) was applied as follows:

p = ((ρ_theo_ − ρ_exp_)/ρ_theo_) · 100% (2)

where p—porosity of the material, %; and ρ_exp_—an experimental value of density of composite, g/cm^3^.

The scanning electron microscope (SEM)—model MIRA3—produced by the Tescan (Brno, Czech Republic), was used in order to assess the structure of the external and internal surface of rotationally molded products. The thin carbon coating with a thickness of approximately 20 nm was deposited on samples using Jeol JEE 4B vacuum evaporator from Jeol USA (Peabody, MA, USA). The structures of the surfaces of the rotationally-molded samples were assessed with an accelerating voltage of 5 kV. The working distance was 10 mm. The secondary electron detector was used.

The tensile strength and elongation at break were estimated following ISO 527 for dumbbell samples type 1BA. Tensile tests were performed on a Zwick/Roell Z020 (Ulm, Germany) apparatus with a cell load capacity of 20 kN at a constant speed of 20 mm/min. At least five specimens were analyzed for each sample.

To determine the crystallization and melting temperatures, as well as the crystalline structure of analyzed composites, DSC analysis was applied. The 5 mg samples were placed in aluminum crucibles with pierced lids. They were heated from 20 to 250 °C with a heating rate of 10 °C/min and then cooled back to the initial temperature with a cooling rate of 10 °C/min. The heating/cooling cycle was performed twice to erase the polymers’ thermal history during the first heating. The measurements were conducted using a Netzsch 204F1 Phoenix apparatus from Netzsch (Selb, Germany) in an inert atmosphere of nitrogen. At least two specimens were analyzed for each sample.

DSC analysis results were also used to calculate the value of the supercooling parameter presented by Qiu et al. [24] according to the following Equation (3):

∆T = T_m_ − T_c_ (3)

where T_m_—melting temperature, °C; and T_c_—crystallization temperature, °C.

Oxidation induction time (OIT) of analyzed composites was determined by the differential scanning calorimetry (DSC) analysis. The 5 mg samples were placed in aluminum crucibles with pierced lids. They were heated from 20 to 190 °C with a heating rate of 20 °C/min in nitrogen, then kept at 190 °C for 5 minutes in nitrogen, and then gas was switched to oxygen, and the time required for sample oxidation was measured. The measurements were conducted using a Netzsch 204F1 Phoenix apparatus from Netzsch (Selb, Germany). At least two specimens were analyzed for each sample.

The thermogravimetric (TGA) analysis of GTR and composites was performed using the TG 209 F3 apparatus from Netzsch (Selb, Germany). Samples of composites weighing approximately 10 mg were placed in a ceramic dish. The study was conducted in an inert gas atmosphere with nitrogen in the range from 30 to 800 °C with a temperature increase rate of 10 °C/min. At least two specimens were analyzed for each sample.

## 3. Results and Discussion

### 3.1. Melt Flow Index of Prepared Composites

The melt flowability is a very important property of polymer materials, since it determines the processing conditions and potential throughput of production processes [25]. In Table 3, there are presented results of the melt flow analysis of prepared composites. As mentioned above, the load of 10 kg was applied due to the low flowability of the WF_100_ sample and type of HDPE matrix. Kazemi-Najafi and Englund [26] also noted the reduction of flowability for partially degraded HDPE, which may simulate recycling process. The use of wood flour often causes the reduction of high-density polyethylene flowability, which was also observed by de Carvalho et al. [27] and Santi et al. [28]. The incorporation of by-products from the food industry resulted in a significant increase in mass and volumetric melt flow index. Such an effect was associated with applied fillers’ chemical composition, particularly with the content of proteins and lipids in coffee silverskin and brewers’ spent grain (see Table 1). These compounds may act as plasticizers for polymer matrix, which noticeably enhances composites’ flowability by reducing melt viscosity [29]. In our previous work, it was shown that MFI values of polycaprolactone/BSG composites were 68 and 138% higher than for polycaprolactone/wheat bran composites, respectively, for 20 and 33 wt% filler loading [30]. Lately, we reported that up to 5 wt% loading of ŁK in high-density polyethylene, and the melt flowability of composites was hardly affected, which is not a typical effect for polymer composites [31]. Similar effects were noted by other researchers [32].

### 3.2. Structure and Physicomechanical Performance of Prepared Composites

**Figure 3:**
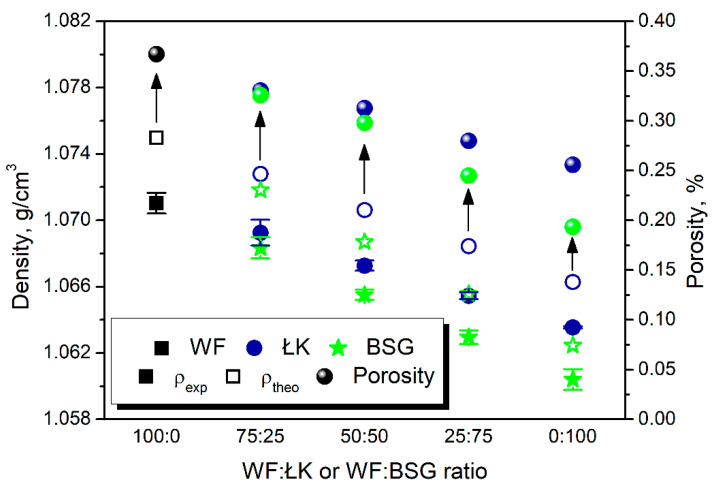
Values of theoretical and experimental density of composites and resulting porosity.

In Figure 3, there are presented values of the experimental and theoretical values of composites density based on the density of neat matrix and applied fillers.

It can be seen that the substitution of wood flour with coffee silverskin and brewers’ spent grain resulted in the slight decrease in composites’ density. It was associated with the lower density of applied waste materials compared to the wood flour. Both ŁK and BSG contain noticeable amounts of proteins (see Table 1), which are characterized by an average density of 1.35 g/cm^3^ (independently of their nature and molecular weight) [33].

Moreover, the theoretical values of composites’ density were higher than measured ones, which indicated the presence of voids in structure. All prepared materials showed relatively low values of porosity below 0.4%, which indicates the good packing of structure during the compression molding. Interestingly, the substitution of the wood flour with coffee silverskin and brewers’ spent grain resulted in the decrease of porosity. Such an effect may result from the higher content of proteins, which may act as plasticizers of polymer matrix [29]. It may enable better encapsulation of filler particles with polyethylene macromolecules reducing void content. However, it does not implicate the better quality and higher strength of the interfacial interactions between matrix and filler particles.

**Figure 4:**
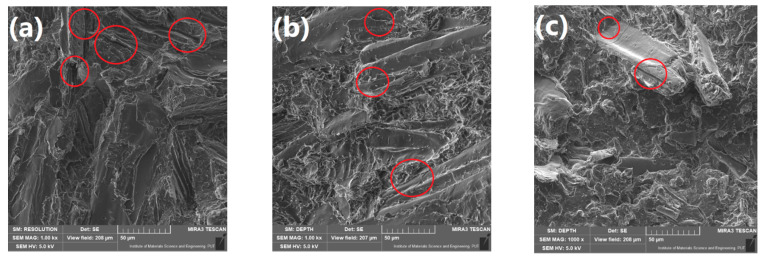

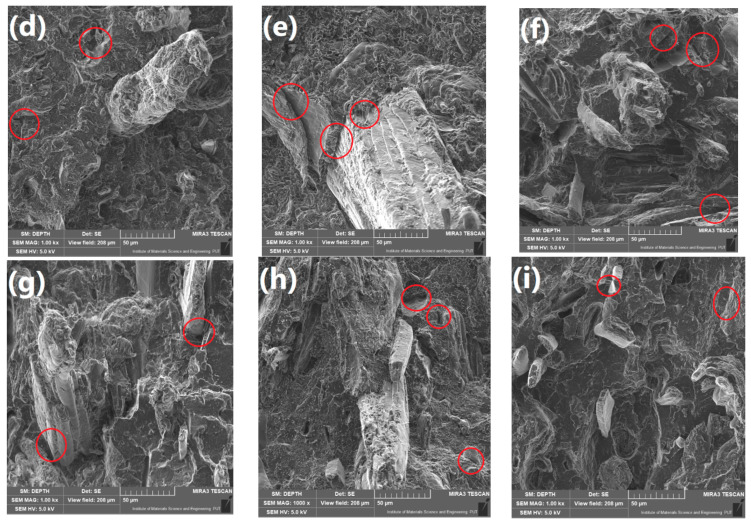
SEM images of brittle fracture areas of samples (**a**) WF_100_, (**b**) WF_75_BSG_25_, (**c**) WF_50_BSG_50_, (**d**) WF_25_BSG_75_, (**e**) WF_0_BSG_100_, (**f**) WF_75_ŁK_25_, (**g**) WF_50_ŁK_50_, (**h**) WF_25_ŁK_25_, and (**i**) WF_0_ŁK_100_ under magnification of ×1000.

For more detailed analysis of composites’ structure, Figure 4 shows the SEM images of brittle fracture areas obtained by breaking samples frozen in liquid nitrogen. All of the prepared composites were characterized by the relatively low values of porosity (see Figure 3), so there were not many voids visible in presented images. The ones detected, either at the matrix-filler interface or between the filler particles, were marked with the red circles. Therefore, the SEM images are in line with the calculated value of porosity, indicating to the efficient realization of melt compounding and compression molding processes.

**Figure 5:**
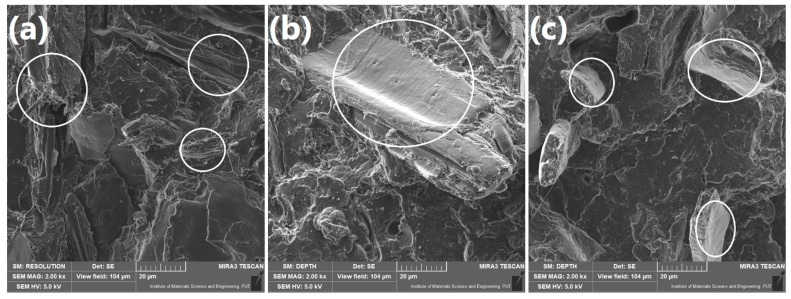
SEM images showing the surface of (**a**) WF, (**b**) BSG, and (**c**) ŁK particles.

It can be clearly seen that the both food-industry by-products showed the noticeably bigger particle size compared to wood flour. Significant differences can be also seen in the appearance of particular fillers. Wood-flour particles are characterized with the layered tube wood-like structures, which was also reported by other researchers [34,35]. Both BSG and ŁK showed more flaked and fibrous structure, which is associated with their origin. These by-products contain husk of barley and inner skin coffee cherry, respectively [36,37]. Figure 5 presents in details the differences in appearance and surface roughness between the particular fillers. Fibrous structure of BSG and ŁK results in the smoother surface of particles and low porosity of the particles [38]. Similar structures were reported in works of Ktenioudaki et al. [39] and Dominici et al. [40]. Such an effect can also significantly affect the mechanical performance of polymer composites, which was repeatedly proven by other researchers [41,42]. Differences in the microstructures between wood and food-industry by-products (pea, bran, and potato fibers) were also noted by Cinelli et al. [10]. They reported fibrous structure of wood flour and flaked, platelet-like particles of other materials.

**Figure 6:**
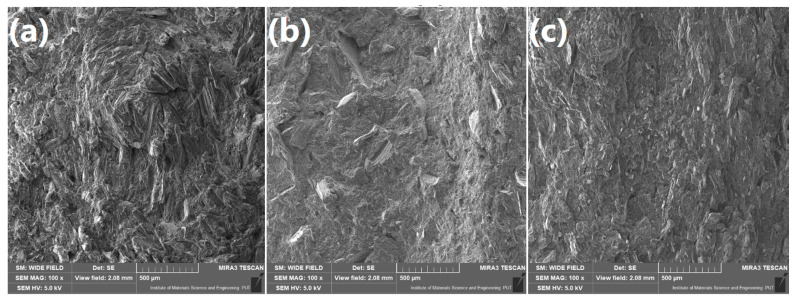
SEM images of brittle fracture areas of samples (**a**) WF_100_, (**b**) WF_0_BSG_100_, and (**c**) WF_0_ŁK_100_ under magnification of ×100.

Moreover, images of brittle fracture areas of samples containing solely WF, BSG, or ŁK presented in Figure 6 point to the significant differences in composites’ roughness. Composites filled with wood flour showed the roughest surface, which was associated to the differences in particle shape and significantly lower particle size and its better distribution in PE matrix. Due to the bigger particle size of BSG and ŁK, in their composites interparticle distance was significantly higher, and the fracture area of polymer phase was smoother. Such an effect may affect the mechanical performance of composites, due to the differences in stress concentration [43].

**Figure 7:**
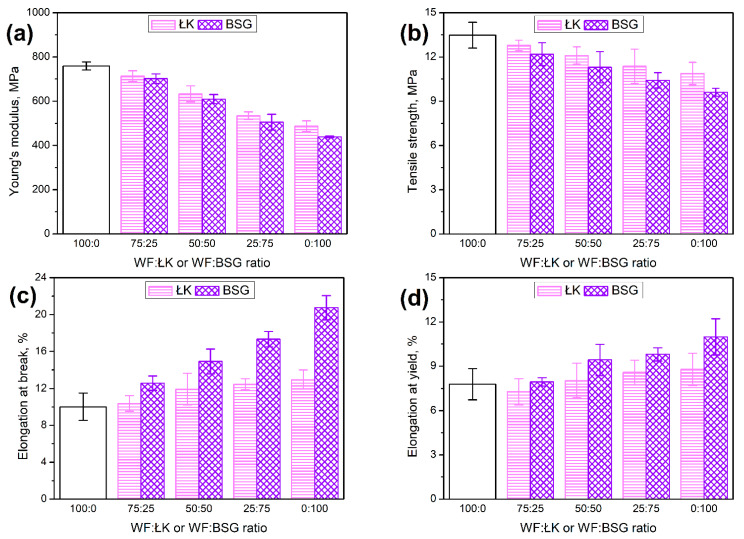
The effect of WF substitution with ŁK and BSG on the (**a**) Young’s modulus, (**b**) tensile strength, (**c**) elongation at break, and (**d**) elongation at yield of prepared composites.

In Figure 7, there are presented results of tensile test of prepared composites. Sample WF100 was used as a reference material. It can be seen that the substitution of the conventional filler with the waste materials from food sector caused the deterioration of the mechanical performance, despite the reduction in porosity. The reduction in tensile strength can be attributed to the differences in particle size and filler distribution in polymer matrix (see Figure 6). Similar observations related to the impact of filler particle size on tensile strength were noted by other researchers [44,45]. They attributed the enhancement of tensile performance with decreasing particle size to the increasing interfacial area between filler and matrix, which enables more efficient stress transfer. Moreover, as presented above (Figure 5), BSG and ŁK fillers are characterized by the significantly smoother surface compared to the WF, which also affects the quality of interface. This effect can be also strengthened by the presence of proteins and lipids, which may act as plasticizers of polymer matrix [46]. Except for the tensile strength, replacement of wood flour with BSG and ŁK affected the composites’ stiffness. The Young’s modulus was decreased from 759 MPa to 633 and 610 MPa, respectively, for 50 wt% loading of coffee silverskin and brewers’ spent grain. The complete replacement of wood flour with waste materials caused 36% and 42% drop of modulus to 487 and 438 MPa, respectively, for ŁK and BSG. Cinelli et al. [10] also noted the deterioration in the tensile performance of composites when potato, pea, or bran fibers were incorporated into composites instead of wood particles. They attributed this effect to the insufficient interfacial adhesion and improper stress transfer when external force is applied.

**Figure 8:**
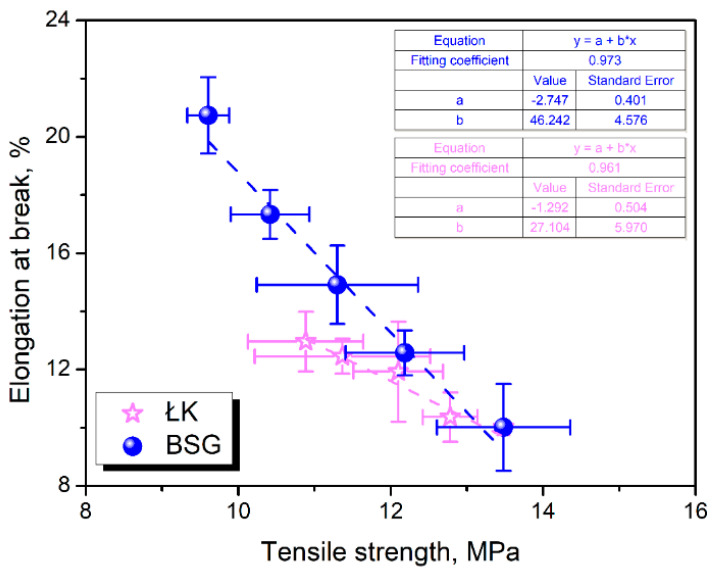
The dependence between tensile strength and elongation at break of prepared composites.

Contrary to the composites’ stiffness and strength, elongation at break was significantly improved by the substitution of wood flour with food-industry by-products. Such an effect can be attributed to the bigger interparticle distance affecting the stress concentration but also to the chemical composition of applied fillers. Moreover, there is a noticeable difference between composites containing ŁK and BSG particles. As mentioned above, the particle size distribution of coffee silverskin was adjusted to match the one of brewers’ spent grain. Therefore, differences can be attributed to the particles’ surface quality and chemical composition. To visualize them, Figure 8 shows the dependence between tensile strength and elongation at break of prepared composites. Significantly higher slope of the line fitted to the experimental data points for BSG indicates the more gradual changes in the composites’ ductility with increasing filler content compared to ŁK. This effect can be attributed to the higher content of proteins and lipids (see Table 1) and the resulting plasticization of polymer matrix. Similar observations were made in our previous paper when BSG was compared to the wheat bran [30]. Zarrinbakhsh et al. [32] compared the performance of composites filled with 25 wt% of coffee silverskin or spent coffee grounds, which differed mostly by the lipids content—5.8 vs. 10.3 wt%. As a result, composites containing coffee silverskin showed 17% higher tensile strength and almost 44% higher modulus. Nevertheless, at the same time, elongation at break was decreased by 35%.

### 3.3. Thermal Properties of Prepared Composites

**Figure 9:**
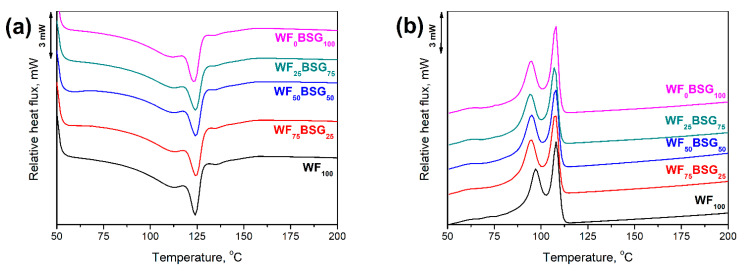

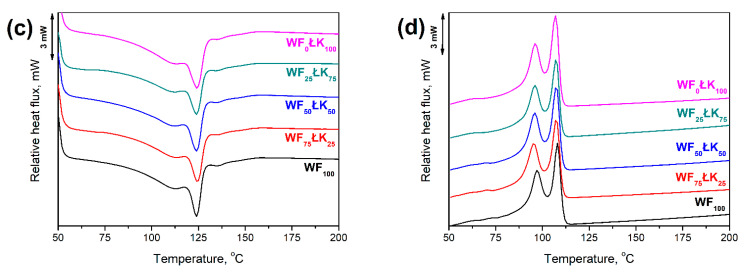
Thermograms obtained for (**a**,**c**) heating and (**b**,**d**) cooling of prepared composites.

Figure 9 and Table 4 presents the results of differential scanning calorimetry results obtained for presented composites. All samples are characterized by the two peaks on melting and crystallization curves. Such an effect indicates inhomogeneity of the polymer phase, which can be attributed to the recycling process or presence of high-density polyethylene fractions in analyzed LDPE stream [47]. Nevertheless, the shape of obtained thermograms points to the relatively low content of high-density polyethylene [48,49].

Substitution of wood flour with food-industry by-products caused the slight decrease in melting and crystallization temperatures of polyethylene phase. Decrease of T_c_ point to the slower crystallization when share of BSG and ŁK were increasing, which can be attributed to the presence of proteins and lipids [40]. Moreover, the supercooling parameter, calculated according to the above-mentioned Formula (1), was increasing with the share of BSG and ŁK. Simultaneous decrease in T_m_ and T_c_, together with the increase in ΔT points to the reduction in nucleating activity of fillers [50]. Such an effect is in line with the differences in particle size, as well as surface area and roughness, between wood flour and food-industry by-products. Small and rough particles of WF may act as nucleating agents more efficiently than smoother and bigger BSG and ŁK [40].

**Figure 10:**
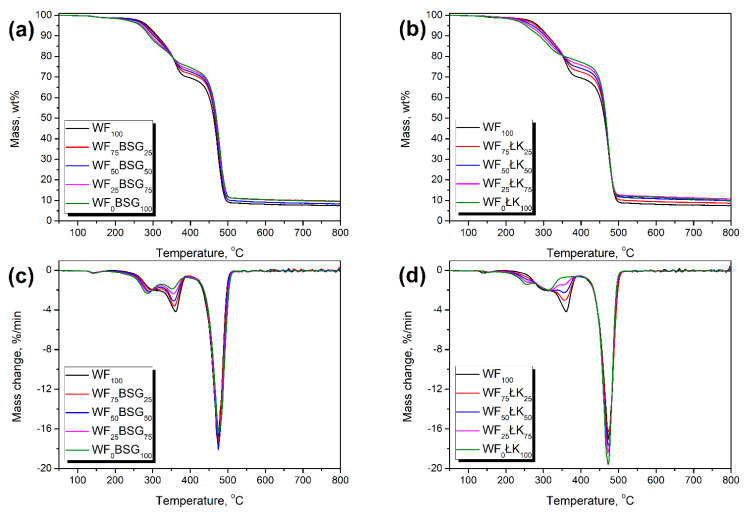
The plots of (**a**,**b**) mass change and (**c**,**d**) differential thermogravimetric curves of prepared composites.

Figure 10 and Table 5 present the results of thermogravimetric analysis of prepared composites. It can be seen that the onset of thermal decomposition is shifted toward lower temperatures when beech wood flour is replaced by the food-industry by-products. Such an effect can be attributed to the differences in fillers’ chemical composition and lower thermal stability of proteins compared to cellulose [51]. Moreover, due to the ash content, resulting from the presence of multiple minerals in BSG and ŁK, the char residue was noticeably increased when the WF was substituted [36,38].

For all samples, the small peak (T_max1_) on DTG curves was noted around 140–155 °C, which could be attributed to the evaporation of residual moisture. Generally, its presence in composites was associated with the hygroscopic character of natural fillers, which was confirmed by other researchers [52,53]. The next peaks, T_max2_, T_max3_, and T_max4_ were related to the decomposition of lignocellulose fillers. Generally, these materials are mainly composed of hemicellulose, cellulose, and lignin. Literature data indicate that the decomposition of hemicellulose and cellulose occurs in the range of 200–370 °C, which is in line with the temperature position of the abovementioned peaks [54,55]. The peaks T_max3_ and T_max4_, present for all composites, and their positions are typical for lignocellulose materials. For WF, the decomposition occurs in three-step manner, attributed to hemicellulose, cellulose, and lignin degradation [21]. Literature works dealing with the thermal stability of beech wood flour report the maximum degradation rates at temperatures which are corresponding with DTG peaks for prepared composites [56,57].

When WF was replaced with brewers’ spent grain, the position of T_max3_ and T_max4_ peaks was shifted toward lower temperatures. Such an effect is attributed to the course of BSG thermal decomposition. In our previous paper [19], we reported thermal stability of the applied filler. It presented two main peaks on DTG curves ~281 and ~341 °C. Therefore, shifts from 312.7 to 285.9 °C and from 360.2 to 352.0 °C were observed. Such results are in line with the literature reports on thermal decomposition of BSG and its composites [58,59,60].

On the other hand, substitution of WF with ŁK filler increased the temperature value of T_max3_ peak. At the same time, the appearance of the peak T_max2_ was noted, as a shoulder peak of T_max3_, when the content of coffee silverskin was increasing. Such an effect can be associated with the course of ŁK thermal decomposition. Sarasini et al. [61] and Totaro et al. [62] also reported the presence of such “shoulder peak” for coffee silverskin around 250–260 °C.

For both food-industry by-products, the shift of T_max4_ peak toward lower temperatures could be attributed to the higher shares of hemicellulose compared to the wood flour (see Table 1) and possibly different crystalline structure of cellulose, which affects thermal stability [63].

The thermal decomposition of lignin occurs at higher temperatures according to the literature data [64]. Therefore, the peak attributed to its degradation was probably overlapped by the peak T_max5_ characteristic for polyolefin decomposition. Its position is typical for polyethylene [65]. According to the literature reports, the decomposition of PE occurs between 300 and 500 °C, with the fastest rate around 460–475 °C [66,67]. Generally, the polyolefins are completely degrading, with the residue usually lower than 1.0 wt% [68].

## 4. Conclusions

In the present paper, we aimed to investigate the impact of conventional beech wood-flour replacement with the two types of food-industry by-products on polyethylene-based composites’ processing and performance. The comprehensive analysis of brewers’ spent grain and coffee silverskin incorporation indicated that they could be efficiently applied in manufacturing of wood–polymer composites. Nevertheless, their application results in the different properties of final material compared to conventional fillers. Composites containing solely BSG and ŁK were characterized by the significantly improved melt flowability. Values of MFR were respectively 227 and 172% higher than for composite filled with beech wood flour. Such an effect should be considered very beneficial for the potential industrial application, because it may enable the increase of production yield. Considering the mechanical performance, replacement of WF with BSG and ŁK improved the ductility of composites, resulting in the gradual increase of elongation at break, especially for brewers’ spent grain. Nevertheless, simultaneous drop of modulus and strength was noted. Such an effect was attributed to the differences in chemical composition of fillers, particularly presence of proteins and lipids, which acted as plasticizers. Composites containing food-industry by-products were also characterized by the lower thermal stability compared to conventional WF. Complete replacement of beech wood flour with brewers’ spent grain and coffee silverskin shifted the onset of decomposition by 31 and 35 °C toward lower temperatures. Nevertheless, its value exceeding 215 °C guarantees safe processing window for polyethylene-based materials.

Concluding, both of the investigated food-industry by-products seem to be very auspicious fillers for polymer composites. They could be introduced as partial replacement of conventional wood flour to engineer materials with desired properties. Further studies associated with these materials should be focused on evaluating changes in processing, structure, mechanical, and thermal properties of composites subjected to accelerated aging tests.

## Figures and Tables

**Table 1:** Literature reports on the composition of applied fillers.

Component	Filler		
	WF	ŁK	BSG
	Content, % Dry Mass		
Cellulose	42.9–47.7	17.9–23.8	16.8–26.0
Hemicellulose	21.4–24.4	7.5–16.7	19.2–29.6
Holocellulose	67.3–69.0	28.6–40.5	40.9–51.5
Lignin	25.5–29.2	28.6–31.0	11.9–27.8
Ash	0.3–0.4	4.5–7.6	1.2–4.6
Protein	-	11.8–18.7	15.3–24.7
Lipids	-	2.1–5.8	3.0–13.0
Ref.	[20,21]	[22]	[23]

**Table 2:** Composition of prepared composites.

Component	Sample				
	WF_100_	WF_75_ŁK_25_ WF_75_BSG_25_	WF_50_ŁK_50_ WF_50_BSG_50_	WF_25_ŁK_75_ WF_25_BSG_75_	WF_0_ŁK_100_ WF_0_BSG_100_
	Content, wt%				
LDPE	60	60	60	60	60
WF	40	30	20	10	0
ŁK/BSG	0	10	20	30	40

**Table 3:** Results of the melt flow analysis of prepared composites.

Sample	MFR, g/10 min	MVR, cm^3^/10 min	Viscosity, Pa·s	Melt density, g/cm^3^
WF_100_	3.230 ± 0.089	3.508 ± 0.076	1.0181	0.927 ± 0.003
WF_75_BSG_25_	4.240 ± 0.071	4.605 ± 0.064	0.7889	0.923 ± 0.003
WF_50_BSG_50_	5.765 ± 0.132	6.273 ± 0.130	0.5751	0.918 ± 0.002
WF_25_BSG_75_	7.565 ± 0.134	8.260 ± 0.156	0.4511	0.914 ± 0.001
WF_0_BSG_100_	10.555 ± 0.316	11.625 ± 0.319	0.3031	0.908 ± 0.002
WF_75_ŁK_25_	4.155 ± 0.070	4.510 ± 0.087	0.7302	0.922 ± 0.002
WF_50_ŁK_50_	5.500 ± 0.349	5.988 ± 0.360	0.5840	0.920 ± 0.007
WF_25_ŁK_75_	6.838 ± 0.300	7.473 ± 0.308	0.4683	0.917 ± 0.005
WF_0_ŁK_100_	8.780 ± 0.426	9.634 ± 0.456	0.3706	0.912 ± 0.002

**Table 4:** The results of differential scanning calorimetry (DSC) analysis of prepared composites.

Sample	T_m1_, °C	T_m2_, °C	T_c1_, °C	T_c2_, °C	ΔT_1_, °C	ΔT_2_, °C
WF_100_	113.0	124.0	96.7	108.0	16.3	16.0
WF_75_BSG_25_	112.9	124.0	95.0	107.9	17.9	16.1
WF_50_BSG_50_	112.9	123.9	95.0	107.8	17.9	16.1
WF_25_BSG_75_	112.8	123.9	94.7	107.6	18.1	16.3
WF_0_BSG_100_	112.4	123.4	94.1	107.0	18.3	16.4
WF_75_ŁK_25_	112.9	124.0	96.2	107.5	16.7	16.5
WF_50_ŁK_50_	112.8	124.0	96.0	107.2	16.8	16.8
WF_25_ŁK_75_	112.7	123.9	96.0	107.1	16.7	16.8
WF_0_ŁK_100_	112.6	123.8	95.8	107.0	16.8	16.8

**Table 5:** The results of thermogravimetric analysis of prepared composites.

Sample	WF_100_	WF_75_BSG_25_	WF_50_BSG_50_	WF_25_BSG_75_	WF_0_BSG_100_	WF_75_ŁK_25_	WF_50_ŁK_50_	WF_25_ŁK_75_	WF_0_ŁK_100_
T_-2%_, °C	250.7	241.7	237.9	222.3	219.2	242.8	233.8	226.3	215.8
T_-5%_, °C	285.1	281.0	277.2	270.4	266.7	280.0	270.9	261.2	252.7
T_-10%_, °C	311.3	307.3	302.2	295.4	290.7	309.9	304.5	297.5	288.8
T_-50%_, °C	462.4	465.3	466.5	467.9	469.5	464.5	465.1	466.2	466.3
Residue, wt%	7.49	7.78	8.42	9.60	9.52	8.74	9.97	10.61	11.08
T_max1_, °C	139.7	139.7	139.8	143.8	146.0	155.4	152.7	152.0	152.1
T_max2_, °C	-	-	-	-	-	-	259.9	258.8	256.9
T_max3_, °C	312.7	298.1	292.2	289.8	285.9	322.2	319.8	314.6	311.0
T_max4_, °C	360.2	357.2	356.3	354.3	352.0	356.9	355.8	351.1	347.8
T_max5_, °C	472.0	474.8	475.0	476.4	476.6	472.6	473.2	473.1	472.5

## Data Availability

Data is contained within the article. The data presented in this study are available in by-products from food industry as a promising alternative for the conventional fillers for wood–polymer composites.

## References

[1] Caldeira C., De Laurentiis V., Corrado S., van Holsteijn F., Sala S. (2019). Quantification of food waste per product group along the food supply chain in the European Union: A mass flow analysis. Resour. Conserv. Recy..

[2] Food Wastage: Key Facts and Figures. http://www.fao.org/news/story/en/item/196402/icode/.

[3] Hejna A., Kosmela P., Formela K., Piszczyk Ł., Haponiuk J.T. (2016). Potential applications of crude glycerol in polymer technology–Current state and perspectives. Renew. Sust. Energy Rev..

[4] Kurek M., Garofulić I.E., Bakić M.T., Ščetar M., Uzelac V.D., Galić K. (2018). Development and evaluation of a novel antioxidant and pH indicator film based on chitosan and food waste sources of antioxidants. Food Hydrocolloid..

[5] Makris D.P., Şahin S. (2019). Polyphenolic Antioxidants from Agri-Food Waste Biomass. Antioxidants.

[6] Badolati N., Masselli R., Maisto M., Di Minno A., Tenore G.C., Stornaiuolo M., Novellino E. (2020). Genotoxicity Assessment of Three Nutraceuticals Containing Natural Antioxidants Extracted from Agri-Food Waste Biomasses. Foods.

[7] Ahmed I.I., Gupta A.K. (2010). Pyrolysis and gasification of food waste: Syngas characteristics and char gasification kinetics. Appl. Energ..

[8] Maag A.R., Paulsen A.D., Amundsen T.J., Yelvington P.E., Tompsett G.A., Timko M.T. (2018). Catalytic Hydrothermal Liquefaction of Food Waste Using CeZrOx. Energies.

[9] Sharma P., Gaur V.K., Kim S.H., Pandey A. (2020). Microbial strategies for bio-transforming food waste into resources. Bioresource Technol..

[10] Cinelli P., Seggiani M., Coltelli M.B., Danti S., Righetti M.C., Gigante V., Sandroni M., Signori F., Lazzeri A. (2021). Overview of Agro-Food Waste and By-Products Valorization for Polymer Synthesis and Modification for Bio-Composite Production. Proceedings.

[11] Cecchi T., Giuliani A., Iacopini F., Santulli C., Sarasini F., Tirillo J. (2019). Unprecedented high percentage of food waste powder filler in poly lactic acid green composites: Synthesis, characterization, and volatile profile. Environ. Sci. Pollut. Res..

[12] Sharma H., Singh I., Misra J.P. (2019). Mechanical and thermal behaviour of food waste (Citrus limetta peel) fillers–based novel epoxy composites. Polym. Polym. Compos..

[13] Lebersorger S., Schneider F. (2011). Discussion on the methodology for determining food waste in household waste composition studies. Waste Manag..

[14] Zhang R., Elmashad H., Hartman K., Wang F., Liu G., Choate C., Gamble P. (2007). Characterization of food waste as feedstock for anaerobic digestion. Bioresour. Technol..

[15] Vieira M.G.A., da Silva M.A., dos Santos L.O., Beppu M.M. (2011). Natural-based plasticizers and biopolymer films: A review. Eur. Polym. J..

[16] Srebrenkoska V., Bogoeva Gaceva G., Dimeski D. (2014). Biocomposites based on polylactic acid and their thermal behavior after recycing. Maced. J. Chem. Chem. Eng..

[17] Tsang Y.F., Kumar V., Samadar P., Yang Y., Lee J., Ok Y.S., Song H., Kim K.H., Kwon E.E., Jeon Y.J. (2019). Production of bioplastic through food waste valorization. Environ. Int..

[18] Hejna A., Formela K. (2019). Sposób Suszenia i Rozdrabniania Młóta Browarnianego. Polish patent.

[19] Hejna A., Barczewski M., Skórczewska K., Szulc J., Chmielnicki B., Korol J., Formela K. (2021). Sustainable upcycling of brewers‘ spent grain by thermo-mechanical treatment in twin-screw extruder. J. Clean. Prod..

[20] Akgul M., Tozluoglu A. (2009). Some Chemical and Morphological Properties of Juvenile Woods from Beech (*Fagus orientalis* L.) and Pine (*Pinus nigra* A.) Plantations. Trend. Appl. Sci. Res..

[21] Bodĭrlǎu R., Teacă C.A., Spiridon I. (2008). Chemical modification of beech wood: Effect on thermal stability. Bioresources.

[22] Hejna A. (2021). Coffee Silverskin as a Potential Bio-Based Antioxidant for Polymer Materials: Brief Review. Proceedings.

[23] Lynch K.M., Steffen E.J., Arendt E.K. (2016). Brewers’ spent grain: A review with an emphasis on food and health. J. I. Brewing.

[24] Qiu W., Zhang F., Endo T., Hirotsu T. (2005). Isocyanate as a compatibilizing agent on the properties of highly crystalline cellulose/polypropylene composites. J. Mater. Sci..

[25] Muhammad Khan R., Mushtaq A., Israr A., Nafees A. (2019). Comparative Study for Melt Flow Index of High Density Polyethylene, Low Density Polyethylene and Linear Low Density Polyethylene. Pakis. J. Eng. Appl. Sci..

[26] Kazemi-Najafi S., Englund K.R. (2013). Effect of highly degraded high-density polyethylene (HDPE) on processing and mechanical properties of wood flour-HDPE composites. J. Appl. Polym. Sci..

[27] De Carvalho M.S., Azevedo J.B., Barbosa J.D.V. (2020). Effect of the melt flow index of an HDPE matrix on the properties of composites with wood particles. Polym. Test..

[28] Santi C.R., Hage E., Vlachopoulos J., Correa C.A. (2009). Rheology and Processing of HDPE/Wood Flour Composites. Int. Polym. Process..

[29] Selmin F., Franceschini I., Cupone I.E., Minghetti P., Cilurzo F. (2015). Aminoacids as non-traditional plasticizers of maltodextrins fast-dissolving film. Carbohydr. Polym..

[30] Hejna A., Formela K., Saeb M.R. (2015). Processing, mechanical and thermal behavior assessments of polycaprolactone/agricultural wastes biocomposites. Ind. Crop. Prod..

[31] Hejna A., Barczewski M., Kosmela P., Mysiukiewicz O., Kuzmin A. (2021). Coffee Silverskin as a Multifunctional Waste Filler for High-Density Polyethylene Green Composites. J. Compos. Sci..

[32] Zarrinbakhsh N., Wang T., Rodriguez-Uribe A., Misra M., Mohanty A.K. (2016). Characterization of Wastes and Coproducts from the Coffee Industry for Composite Material Production. BioResources.

[33] Fischer H., Polikarpov I., Craievich A.F. (2009). Average protein density is a molecular-weight-dependent function. Protein Sci..

[34] Yin B., Hakkarainen M. (2013). Green Plasticizers from Liquefied Wood. Waste Biomass Valori..

[35] Klímek P., Wimmer R., Kumar Mishra P., Kúdela J. (2017). Utilizing brewer’s-spent-grain in wood-based particleboard manufacturing. J. Clean. Prod..

[36] Hejna A. (2021). Potential applications of by-products from the coffee industry in polymer technology—Current state and perspectives. Waste Manag..

[37] Ikram S., Huang L., Zhang H., Wang J., Yin M. (2017). Composition and Nutrient Value Proposition of Brewers Spent Grain. J. Food Sci..

[38] Mussatto S.I., Dragone G., Roberto I.C. (2006). Brewers’ spent grain: Generation, characteristics and potential applications. J. Cereal Sci..

[39] Ktenioudaki A., Chaurin V., Reis S.F., Gallagher E. (2012). Brewer’s spent grain as a functional ingredient for breadsticks. Int. J. Food Sci. Technol..

[40] Dominici F., García García D., Fombuena V., Luzi F., Puglia D., Torre L., Balart R. (2019). Bio-Polyethylene-Based Composites Reinforced with Alkali and Palmitoyl Chloride-Treated Coffee Silverskin. Molecules.

[41] Marghalani H.Y. (2010). Effect of filler particles on surface roughness of experimental composite series. J. Appl. Oral Sci..

[42] Chattopadhyay P.K., Das N.C., Chattopadhyay S. (2011). Influence of interfacial roughness and the hybrid filler microstructures on the properties of ternary elastomeric composites. Compos. Part A Appl. Sci. Manuf..

[43] Njoku R.E., Okon A.E., Ikpaki T.C. (2011). Effects of Variation of Particle Size and Weight Fraction on the Tensile Strength and Modulus of Periwinkle Shell Reinforced Polyester Composite. Niger. J. Technol..

[44] Durowaye S.I., Lawal G.I., Olagbaju O.I. (2014). Microstructure and Mechanical Properties of Sisal Particles Reinforced Polypropylene Composite. Int. J. Compos. Mater..

[45] Hassan S.B., Aigbodion V.S., Patrick S.N. (2012). Development of Polyester/Eggshell Particulate Composites. Tribol. Ind..

[46] Stein T.M., Gordon S.H., Greene R.V. (1999). Amino acids as plasticizers: II Use of quantitative structure-property relationships to predict the behavior of monoammoniummonocarboxylate plasticizers in starch-glycerol blends. Carbohydr. Polym..

[47] Fonseca C.A., Harrison I.R. (1998). An investigation of co-crystallization in LDPE/HDPE blends using DSC and TREF. Thermochim. Acta.

[48] Munaro M., Akcelrud L. (2007). Correlations between composition and crystallinity of LDPE/HDPE blends. J. Polym. Res..

[49] Li D., Zhou L., Wang X., He L., Yang X. (2019). Effect of Crystallinity of Polyethylene with Different Densities on Breakdown Strength and Conductance Property. Materials.

[50] Hejna A., Kosmela P. (2020). Insights into Compatibilization of Poly(ε-caprolactone)-based Biocomposites with Diisocyanates as Modifiers of Cellulose Fillers. Mindanao J. Sci. Technol..

[51] Li C., Luo J., Qin Z., Chen H., Gao Q., Li J. (2015). Mechanical and thermal properties of microcrystalline cellulose-reinforced soy protein isolate–gelatin eco-friendly films. RSC Adv..

[52] Almeida G., Remond R., Perre P. (2018). Hygroscopic behaviour of lignocellulosic materials: Dataset at oscillating relative humidity variations. J. Build. Eng..

[53] George J., Sreekala M.S., Thomas S. (2001). A review on interface modification and characterization of natural fiber reinforced plastic composites. Polym. Eng. Sci..

[54] Shen D.K., Gu S. (2009). The mechanism for thermal decomposition of cellulose and its main products. Bioresour. Technol..

[55] Bogoeva-Gaceva G., Dimeski D., Srebrenkoska V. (2013). Biocomposites based on poly (lactic acid) and kenaf fibers: Effect of micro-fibrillated cellulose. Maced. J. Chem. Chem. Eng..

[56] Vargun E., Baysal E., Turkoglu T., Yuksel M., Toker H. (2019). Thermal degradation of oriental beech wood impregnated with different inorganic salts. Maderas. Cie. Tecnol..

[57] Marková I., Ladomerský J., Hroncová E., Mračková E. (2018). Thermal parameters of beech wood dust. BioResources.

[58] Vanreppelen K., Vanderheyden S., Kuppens T., Schreurs S., Yperman J., Carleer R. (2014). Activated carbon from pyrolysis of brewer’s spent grain: Production and adsorption properties. Waste Manag. Res..

[59] Borel L.D.M.S., Lira T.S., Ribeiro J.A., Ataíde C.H., Barrozo M.A.S. (2018). Pyrolysis of brewer’s spent grain: Kinetic study and products identification. Ind. Crop. Prod..

[60] Zedler Ł., Colom X., Saeb M.R., Formela K. (2018). Preparation and characterization of natural rubber composites highly filled with brewers’ spent grain/ground tire rubber hybrid reinforcement. Compos. Part B Eng..

[61] Sarasini F., Tirillò J., Zuorro A., Maffei G., Lavecchia R., Puglia D., Dominici F., Luzi F., Valente T., Torre L. (2018). Recycling coffee silverskin in sustainable composites based on a poly(butylene adipate-co-terephthalate)/poly(3-hydroxybutyrate-co-3-hydroxyvalerate) matrix. Ind. Crop. Prod..

[62] Totaro G., Sisti L., Fiorini M., Lancellotti I., Andreola F.N., Saccani A. (2019). Formulation of Green Particulate Composites from PLA and PBS Matrix and Wastes Deriving from the Coffee Production. J. Polym. Environ..

[63] Sharma A., Mandal T., Goswami S. (2018). Cellulose nanofibers from rice straw: Process development for improved delignification and better crystallinity index. Trends Carbohyd. Res..

[64] Zhang N., Tao P., Lu Y., Nie S. (2019). Effect of lignin on the thermal stability of cellulose nanofibrils produced from bagasse pulp. Cellulose.

[65] Aboulkas A., El Harfi K., El Bouadili A. (2010). Thermal degradation behaviors of polyethylene and polypropylene. Part I: Pyrolysis kinetics and mechanisms. Energ. Conv. Manage..

[66] Das P., Tiwari P. (2018). Valorization of packaging plastic waste by slow pyrolysis. Resour. Conserv. Recy..

[67] Rego A., Silva A.S., Grillo A.V., Santos B.F. (2019). Thermogravimetric Study of Raw and Recycled Polyethylene Using Genetic Algorithm for Kinetic Parameters Estimation. Chem. Eng. Trans..

[68] Contat-Rodrigo L., Ribes-Greus A., Imrie C.T. (2002). Thermal analysis of high-density polyethylene and low-density polyethylene with enhanced biodegradability. J. Appl. Polym. Sci..

